# Examining the trend of mortality of genitourinary system cancers in Babol, North Iran (2013–2021)

**DOI:** 10.1186/s12894-024-01523-7

**Published:** 2024-07-30

**Authors:** Pouyan Ebrahimi, Mohsen Karami, Seyed-Hossein Hosseini-Berneti, Amir-Hossein Lashkarbolouki, Sana Keshtegar, Mohammad-Amin Ghezel, Hossein-Ali Nikbakht

**Affiliations:** 1 Student Research Committee, Babol University of Medical Sciences, Babol, Iran; 2 Infectious Diseases and Tropical Medicine Research Center, Health Research Institute, Department of Parasitology and Mycology, Babol University of Medical Sciences, Babol, Iran; 3 Social Determinants of Health Research Center, Health Research Institute, Babol University of Medical Sciences, Babol, 47176-47745 Iran

**Keywords:** Genitourinary cancers, Iran, Mortality, Trends

## Abstract

**Background:**

Cancers of the genitourinary system, particularly prostate, bladder, and kidney cancer, exhibit a high prevalence. Consequently, predicting the morbidity and mortality of genitourinary cancers holds great significance for future planning and implementation. This study aimed to examine the crude and age-standardized rates of mortality and the trend of genitourinary cancers over nine years in northern Iran.

**Methods:**

This cross-sectional study used data on the number of deaths attributed to genitourinary cancers recorded in Babol City between 2013 and 2021 through the cause of death registration and classification system. Population estimates were derived from the latest census reports. Subsequently, crude and age-standardized rates, as well as trends for genitourinary cancers, were calculated.

**Results:**

A total of 307 deaths occurred, with an average age of 75.6 ± 14.3 years due to genitourinary cancers. The crude and age-standardized rates of genitourinary cancers increased from 2.7 and 1.9 per hundred thousand people in 2013 to 7.7 and 5.9 per hundred thousand people in 2021, respectively. Over the study period, death rates significantly rose for men (*P* < 0.001) and remained constant for women (*P* = 0.444). Examination of genitourinary cancers revealed an upward trend for bladder (*P* = 0.012) and prostate (*P* = 0.012) cancers, while a stable trend was observed for kidney (*P* = 0.070) and testicular (*P* = 0.139) cancers.

**Conclusions:**

The age-standardized rate and trend of genitourinary cancers are rising. Consequently, this study emphasizes the importance of prevention through screening programs, raising awareness, and utilizing appropriate diagnostic methods.

## Introduction

Cancer is one of the leading causes of death in developed and less developed countries. The formation of tumors results from the uncontrolled growth and proliferation of cells in specific areas of the body, and their transformation into malignant cells can lead to cancer development [1]. In 2020, the global estimate indicated approximately 19.3 million new cancer cases and nearly 10.0 million cancer-related deaths [2]. In Iran, cancer stands as the second leading cause of death following accidents and disasters [3]. According to statistics from the World Health Organization (WHO) 2020, Iran has reported more than 130,000 new cases and approximately 80,000 deaths from various types of cancer [2].

Genitourinary cancers encompass malignancies that develop in various parts of the genitourinary system, affecting both men and women, albeit with differing incidence and mortality rates between the sexes [4]. Genitourinary tract cancers, especially prostate, bladder, and kidney cancers, are among the most common cancers worldwide [4, 5]. According to GLOBOCAN estimates, prostate cancer accounts for 14.1% of malignant neoplasms in men and 6.8% of cancer-related deaths, ranking as the fifth most common cause. Bladder cancer is responsible for 2.1% of all cancer-related deaths in both sexes, with 440,864 new cases in men and 132,414 in women. Kidney cancer is more prevalent in men, with 271,249 new cases, compared to 160,039 in women. Other genitourinary cancers, such as testicular cancer and penile cancer, are less common, representing approximately 0.4% and 0.2% of new cases, respectively. Each accounts for approximately 0.1% of cancer-related deaths [2].

The symptoms of genitourinary cancers vary depending on the type and location of the cancer, and diagnosis is performed based on the type and location using ultrasound, endoscopy, and biopsy [6]. Treatment methods for urogenital cancers include surgery, radiation therapy, chemotherapy, hormone therapy, and other alternative methods, which are prescribed depending on the type and stage of cancer [7]. Prevention and early diagnosis of these cancers can lead to better treatment results and longer patient survival [8]. Age, gender, family history, high-risk behaviors such as smoking and unhealthy diet play crucial roles in increasing the risk of genitourinary cancers. Identifying and controlling these risk factors can effectively prevent and reduce the incidence of these cancers [8].

Investigating the trend of the cancer mortality rate is particularly important, and analyzing it will help health systems allocate appropriate public health priorities and resources. Additionally, examining the trend of mortality data is crucial for evaluating the effectiveness of preventive and therapeutic measures. It serves as an important indicator for investigating the causes of diseases in epidemiological studies [9]. In Iran, the Civil Registration and Vital Statistics (CRVS) system is active in 30 provinces, recording and categorizing deaths based on their causes. It is the best data source for estimating cancer mortality [10].

Babol City, with a population of more than five hundred thousand people, is one of the most populated cities in northern Iran. Given the significance of genitourinary cancers in public health and the observed changes in the incidence and mortality rates of these cancers in recent years, this study aims to investigate the trends in mortality of genitourinary cancers and compare the results with national and international statistics over a 9-year period (2013 to 2021) in Babol.

## Methods

In this cross-sectional study, all deaths registered due to genitourinary cancer in Babol, based on the death registration and classification system affiliated with Babol University of Medical Sciences, during a 9-year period from 2013 to 2021, were examined. This research was approved by the code of ethics (MUBABOL.HRI.REC.1401.153).

Sources of death data included valid death certificates from funeral homes, hospitals, forensic medicine, and other cases. The quality of this information was also reviewed by relevant experts through a qualitative examination of deaths caused by genital cancer. This review aimed to identify and address issues such as duplicate case records, improbable causes of death concerning gender and age, incorrect coding definitions, and other potential errors. Additionally, the information was further validated by re-examining the records associated with each case. After removing duplicate entries, minimizing data recording errors, and receiving approval from the Ministry of Health officials, the data were deemed reliable for reporting in our study.

The causes of death were coded according to the tenth edition of the International Statistical Classification of Diseases and Related Health Problems (ICD-10) [11]. The codes for the genitourinary system from top to bottom [12] and their division by GLOBOCAN 2020 [13] are as follows: adrenal cancer (C74), bladder cancer (C67), kidney cancer (C64-65), penile cancer (C60), prostate cancer (C61), and testicular cancer (C62). Since adrenal cancer is not included in the GLOBOCAN 2020 classifications and there have been no reports of penile cancer in Babol City from 2013 to 2021, these cancers were excluded from the study. It should also be noted that genitourinary system cancers include kidney and bladder cancers in both sexes and prostate and testicular cancers only in men [12].

To calculate and compare cancer mortality rates, it was necessary to estimate the county’s population from 2013 to 2021. In Iran, censuses are conducted every five years, with the most recent ones occurring in 2011 and 2016. However, due to the COVID-19 pandemic, the census scheduled for 2021 was not conducted. Consequently, the populations for the years under study were estimated based on the population growth observed between the 2011 and 2016 censuses. The population of Babol County in the 2016 census was recorded as 531,930.

The average annual population growth rate was calculated using the following formula.

$$r = \;\sqrt[{{}^n}]{{\frac{{pn}}{{p0}}}} - 1$$

Additionally, to estimate the population for different age groups from 2013 to 2021, we used the differences in the age groups from the census years and the estimated population.

The data were analyzed using STATA version 14 software. Qualitative data are presented as frequency and percentage, while mean and standard deviation were used to express quantitative data. Using the census data of the Iran Statistics Center, the city’s population was analyzed by age group to calculate the death rates. The global standard population of the International Agency for Research on Cancer (IARC) and the standard population presented in GLOBOCAN were utilized to calculate the age-standardized rate (ASR) using the direct method per hundred thousand people. Additionally, crude and age-standardized rates with 95% confidence intervals were reported. Finally, according to the mentioned factors, the Cochran–Armitage test for trend was used to determine the trend of mortality during the investigated years in this study. The level of significance was set at *P* < 0.05. Also, to examine the trend of crude mortality rate for different years, joinpoint regression was used based on the log-linear model. The analysis of the trend was carried out using Joinpoint Regression Program 4.9.1.0.

## Results

In this study, out of the total 3,294 cancer-related deaths in Babol from 2013 to 2021, 307 (9.3%) patients were attributed to genitourinary cancer. Among these cases, 286 (93.2%) patients were male, and 145 (55.6%) were residents of the city. Prostate cancer was the most prevalent cancer, accounting for 213 (69.4%) deaths, followed by bladder cancer with 62 (20.2%), kidney cancer with 22 (7.2%), and testicular cancer with 10 (3.3%) deaths.

In general, the average age of the patients was 75.6 ± 14.3 years, ranging from 1 to 98 years, with 290 (94.5%) cases being over 50 years old. The highest mean age was observed for patients with prostate cancer (79.5 ± 9.2 years) and bladder cancer (72.6 ± 13.1 years), while the lowest mean age was noted for patients with testicular cancer (22.9 ± 1.43 years) and kidney cancer (60.8 ± 21.9 years).

**Fig. 1:**
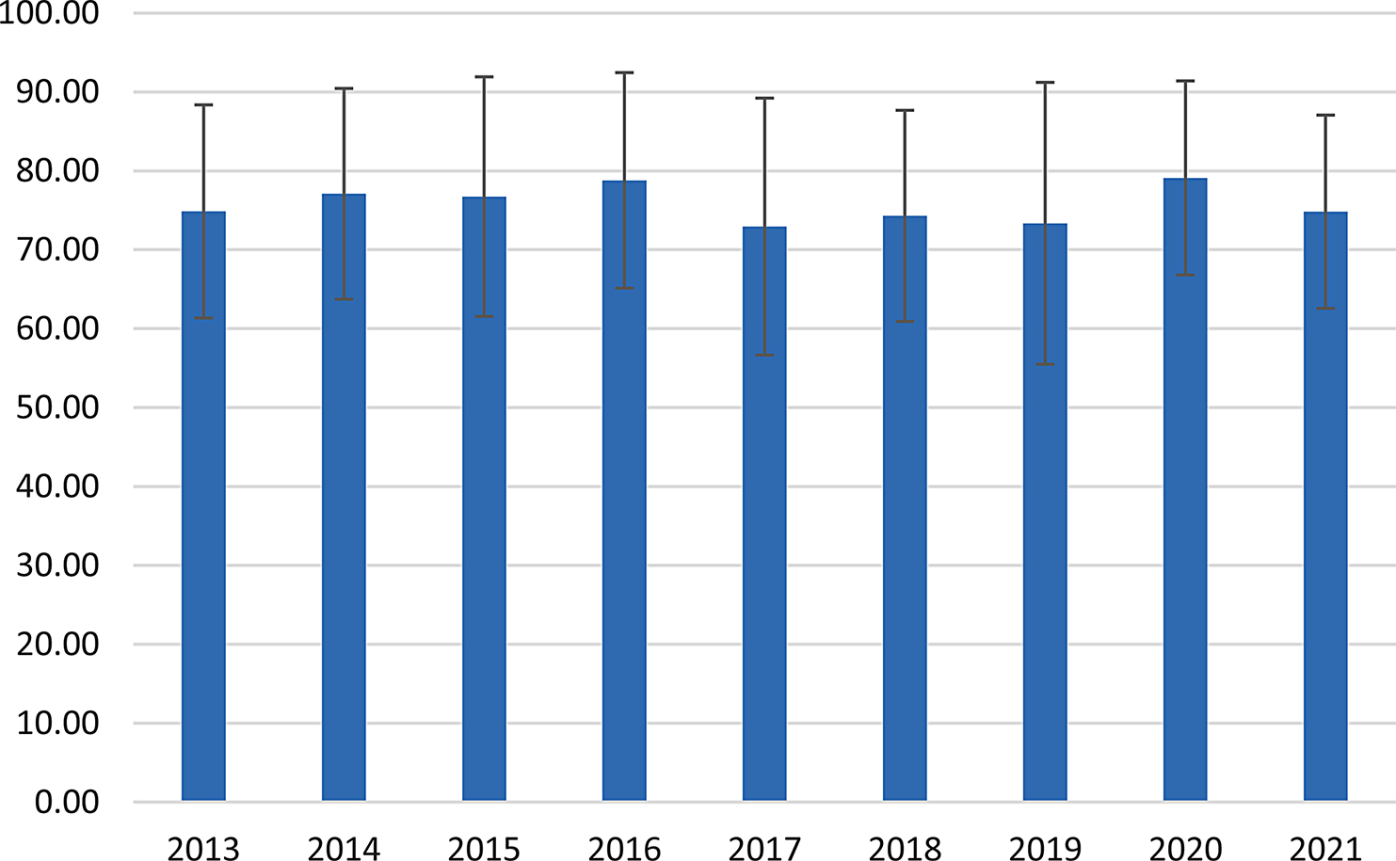
Mean (standard deviation) age of genitourinary system cancers in the studied years

**Fig. 2:**
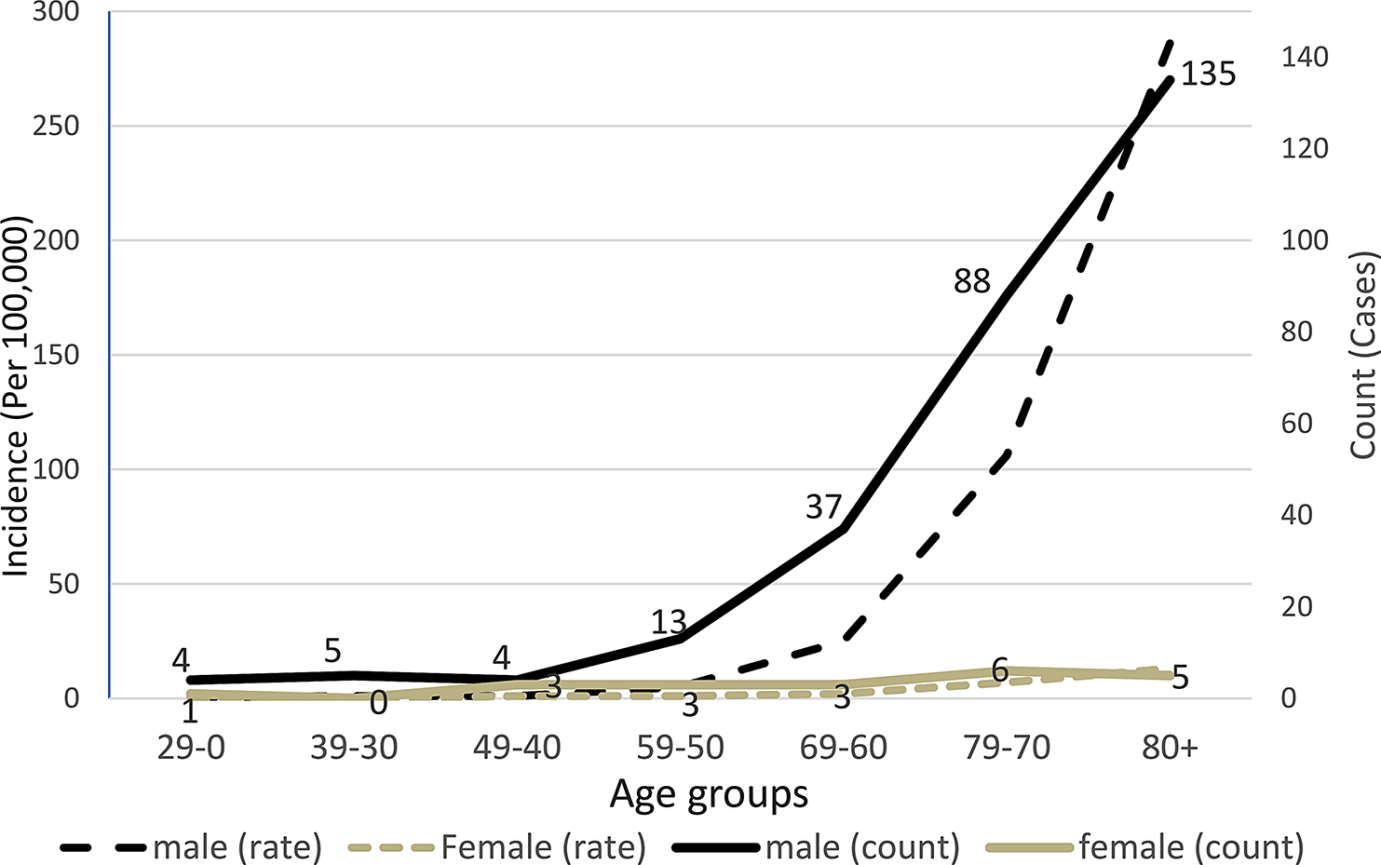
Examining the trend, the number of deaths, and the incidence of death per hundred thousand population due to the genitourinary system cancers according to age groups in Babol (2013–2021) (P-value Trend < 0.001 for men and P-value Trend < 0.001 for women)

The average age of genitourinary system cancers by year is depicted in Fig. 1. A continuous increase in the number of deaths by sex and age group was observed in genitourinary system cancers, and this increasing trend was significant for both sexes (*P* < 0.05 for both) (Fig. 2).

**Fig. 3:**
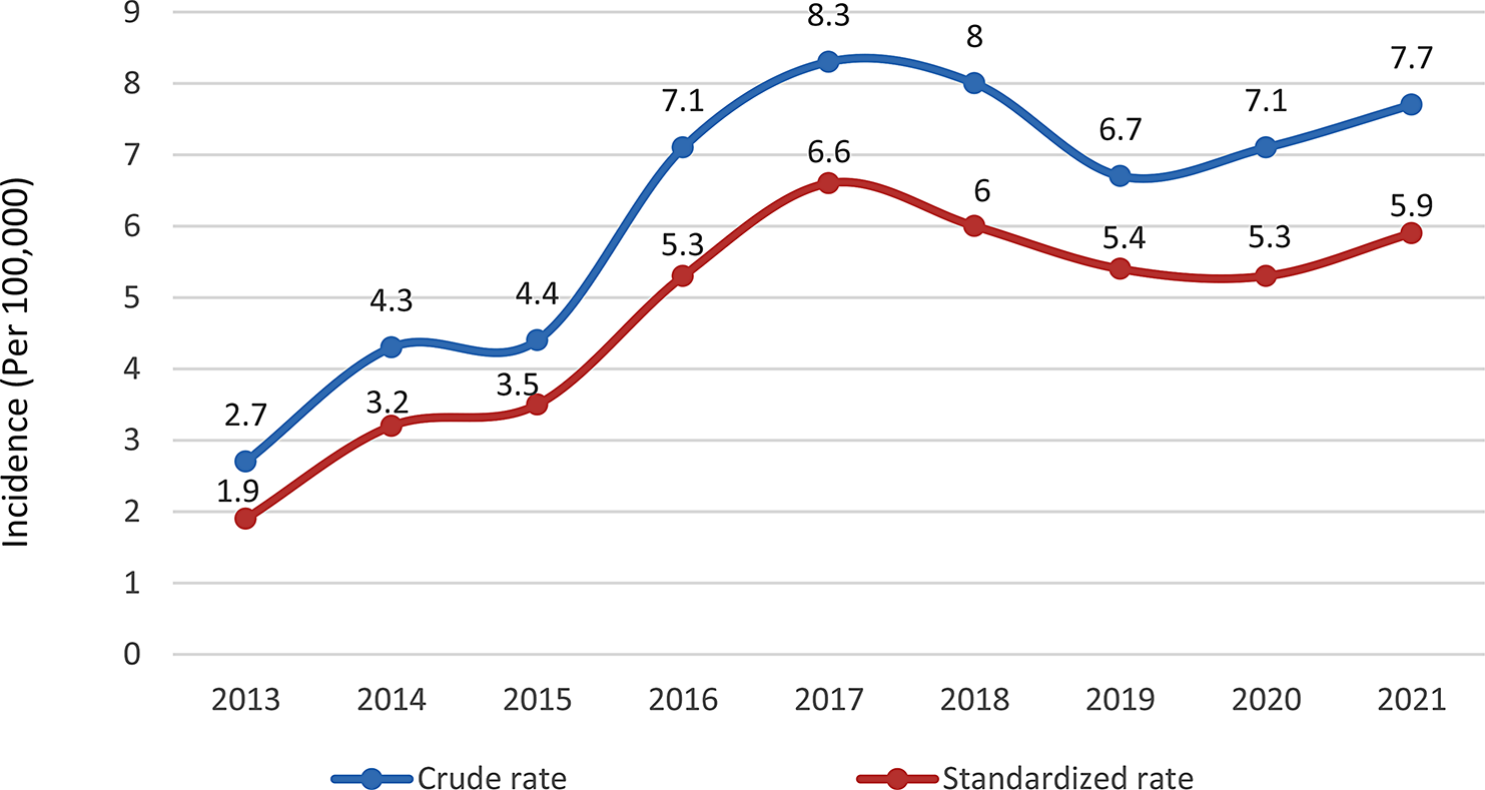
Crude and age-standardized trends in the mortality rate of genitourinary system cancers per hundred thousand people in Babol (2013–2021)

The crude mortality rate in all patients was greater than the age-standardized mortality rate for genitourinary system cancers. The crude and age-standardized mortality rates of genitourinary cancers in 2013 were 2.7 and 1.9, respectively, which increased to 7.7 and 5.9 per 100,000 people in 2021, showing a significant increasing trend (*P* < 0.001) (Fig. 3). In a more detailed examination of the mortality rate of genitourinary system cancers according to sex and year, 2017 for men and 2018 for women had the highest crude mortality rate and age-standardized mortality rate, respectively. The lowest death rate for men was in 2013, and for women, it was in 2014 (Table 1).

**Table 1:** Crude and age-standardized mortality rates of genitourinary system cancers by the studied years and gender per hundred thousand people in Babol (2013–2021)

Years	Male			Female		
	Crude death rate	Age- standardized mortality rate		Crude death rate	Age- Standardized mortality rate	
		Rate	95% confidence interval		Rate	95% confidence interval
2013	4.7	3.1	1.3–4.9	0.8	0.6	0.0–1.5
2014	8.5	6.2	3.5–8.9	0.0	0.0	0.0–0.0
2015	8.0	6.3	3.5–9.2	0.8	0.5	0.0–1.1
2016	13.1	9.1	5.9–12.3	1.1	1.0	0.0–2.2
2017	15.8	12.2	8.4–16.0	0.7	0.8	0.0–2.0
2018	14.2	9.9	6.7–13.2	1.8	1.7	0.2–3.2
2019	12.2	10.0	6.4–13.5	1.1	0.7	0.0–1.6
2020	13.8	10.0	6.7–13.3	0.4	0.2	0.0–0.7
2021	14.3	10.7	7.3–14.2	1.1	0.9	0.0–2.0

*P-value trend for male < 0.001 and female = 0.444

### The mortality trend of genitourinary system cancers

#### Prostate cancer

In examining the mortality trend of prostate cancer, the crude and age-standardized mortality rates in 2013 were 3.9 and 2.7, respectively, which increased to 9.4 and 6.8 per 100,000 people in 2021, showing a significant increasing trend (*P* = 0.012). Aside from that, the crude and age-standardized mortality rates for prostate cancer were the highest in 2017 (11.4 and 8.7 per 100,000, respectively) (Table 2).

**Table 2:** The trend of crude and age-standardized mortality rates by the studied years for each of the genitourinary cancers per one hundred thousand people in Babol (2013–2021)

Years	Rate	Bladder	Kidney	Prostate	Testis
2013	Crude	0.8	0	3.9	0
	Standardized	0.5	0	2.7	0
2014	Crude	0.6	0.2	6.9	0
	Standardized	0.5	0.2	5.0	0
2015	Crude	1.0	0.2	6.1	0.4
	Standardized	0.7	0.1	4.6	0.5
2016	Crude	0.8	0.6	10.8	0.7
	Standardized	0.6	0.5	7.4	0.7
2017	Crude	1.3	1.1	11.4	0.4
	Standardized	1.1	1.0	8.7	0.2
2018	Crude	1.8	0.4	11.3	0.4
	Standardized	1.5	0.3	7.8	0.2
2019	Crude	2.0	0.4	8.2	0.4
	Standardized	1.7	0.4	6.0	0.5
2020	Crude	1.4	0.4	9.9	0.7
	Standardized	1.2	0.3	6.7	0.5
2021	Crude	1.8	0.9	9.4	0.7
	Standardized	1.4	0.8	6.8	0.5
P-trend		0.012	0.070	0.012	0.139

#### Bladder cancer

The crude and age-standardized mortality rates for bladder cancer were 0.8 and 0.5 in 2013, respectively, rising to 1.8 and 1.4 per 100,000 people in 2021, indicating an increasing trend (*P* = 0.012). Besides that, the crude and age-standardized mortality rates for bladder cancer were the highest in 2019 (2.0 and 1.7 per 100,000, respectively) (Table 2).

#### Kidney cancer

Kidney cancer mortality rates in 2013 were zero. The crude and age-standardized mortality rates for kidney cancer have both increased to 0.9 and 0.8 in 2021, respectively, but this increasing trend was not statistically significant (*P* = 0.070) (Table 2).

#### Testicular cancer

In 2013, the crude and age-standardized mortality rates for kidney cancer were both zero. In 2021, these rates increased to 0.7 and 0.5, respectively, but this trend was not statistically significant (*P* = 0.139) (Table 2).

According to the Joinpoint regression analysis, the 9-year trend of the YLL rate due to premature mortality was increasing in males, with the annual percent change (APC) being 12.1% (95% CI 3.5 to -21.3, *p* = 0.011), and stable for females, with the APC being 25.2% (95% CI -21.5 to 99.5, *p* = 0.292). The model did not show any joinpoint; hence, the AAPC (Average Annual Percent Change) is the same as the APC.

## Discussion

In this study, a report was compiled on the mortality rate and trend of different types of genitourinary cancer in men and women of Babol City from 2013 to 2021. Prostate cancer has been the most common cancer of the genitourinary system, constituting 4.69% of recorded cancer deaths over this 9-year period in Babol. An examination of the number of deaths by sex and age groups revealed a continuous increase in genitourinary system cancers, and this upward trend was significant in both sexes. Among the patients in our study, the highest average age was observed for prostate cancer (79.5 ± 9.2 years) and bladder cancer (72.6 ± 13.1 years), respectively. The crude mortality rate in all patients was greater than the age-standardized rate for genitourinary system cancers. In a more detailed analysis of the mortality rate of genitourinary system cancers based on gender and year, 2017 for men and 2018 for women had the highest crude mortality rate and age-standardized mortality rate, respectively. The lowest death rate for men was recorded in 2013, and for women, it was in 2014.

In Babol, the incidence rate of prostate cancer was 6.5 per hundred thousand people, which differs from the rates reported in Iran (21.2) [2] and globally (31.2) [14]. Shafiei et al.‘s study, which compared the data from this research with global prostate cancer statistics, revealed an incidence rate ranging from 6.3 to 83.4 per 100,000 men in different regions [3]. The highest rates were observed in Western Europe and North American countries, while the lowest rates were observed in Asia and North Africa [15]. Risk factors for prostate cancer include increasing age, a family history of the disease, and lifestyle factors such as smoking, obesity, and nutritional status [16–18]. Prostate cancer screening involves digital rectal examination (DRE), prostate-specific antigen (PSA) blood test, and transrectal ultrasound-guided sampling (TRUS) [19–21]. Treatment options include radiotherapy, chemotherapy, and hormone therapy. Although there are suggestions for lifestyle or dietary changes, there are currently no proven prevention methods for prostate cancer [21].

Kidney cancer had a frequency of 0.7% in our study (excluding the pelvis). This cancer is more prevalent in men than women, with its incidence and mortality rate in men being almost four times greater than those in women globally [22]. The highest incidence rates for both sexes are in Southern Europe (Greece, Spain, and Italy), Western Europe (Belgium and the Netherlands) and North America. However, the highest global rate among women is in Hungary [23]. In Iran, 5065 new cases and 1760 deaths due to this malignancy were recorded in 2020 [24].

The most common type of bladder cancer is the urothelial type. Squamous cell cancer and adenocarcinoma are less common (5% and 2%, respectively) and are associated with a more advanced stage of the disease and higher mortality [24]. In our study, the frequency of bladder cancer in Babol over these nine years was estimated to be 1.9%. Risk factors for bladder cancer include smoking, exposure to aniline dyes, aromatic amines, and polycyclic aromatic hydrocarbons, chronic urinary tract infections, chronic use of urinary catheters, bladder stones, history of pelvic radiotherapy, and chemotherapy [25]. The most common symptom of bladder cancer is severe and painless hematuria, occurring in 80% of affected patients [26]. Treatment involves chemotherapy and radical cystectomy [27], but there is still a risk of disease recurrence.

Testicular cancer does not exhibit a high mortality rate, and the reported Age-Standardized Rate (ASR) is approximately, which aligns with the rate found in our study [28]. Although testicular cancer is one of the least common cancers, its incidence appears to be increasing globally. The incidence of this disease is greater in developed countries than in developing ones, and in recent years the incidence of this disease has increased in the United States and several other countries [4, 29, 30]. Possible risk factors for this cancer include an undescended testicle, family history, and infection [31]. Current treatments for this disease have shown favorable survival outcomes [32].

One limitation of this study is the absence of certain variables, such as job status, education, and place of residence. Future research should focus on more accurate recording of these variables, among others. Another potential limitation is the underestimation of cancer mortality due to poor registration, as well as the failure to detect cancer as a source of bias. Patients who present to a doctor in the advanced stages of the disease and who die without a diagnosis, fall into this category. Further studies could explore underlying factors such as smoking and nutrition in more detail to provide a comprehensive understanding. The strengths of this study include reporting the standardized rates of each genitourinary cancer, along with their trends, over a 9-year period.

## Conclusions

In examining the trends in the mortality of patients with genitourinary system cancers, the standardized rates for bladder and prostate cancer have increased significantly. Although the crude mortality rates and Age-Standardized Mortality Rates (ASMR) of kidney and testicular cancers increased in the years after 2016 compared to before, they generally followed a steady trend. In light of the importance of cancer trends and cancer-related mortality in Babol, it is imperative to establish a detailed follow-up plan for each type of cancer and investigate the causes and risk factors influencing patient mortality. Additionally, implementing proper screening programs alongside public awareness campaigns about the risk factors for different cancers in this city can contribute to reducing the death rate and disease burden in the population of Babol. Our study can aid in designing a monitoring program and assessing the risk factors associated with cancers.

## Data Availability

The datasets used and analyzed during the current study are available from the corresponding author or Health Deputy at the Babol University of Medical Sciences upon reasonable request.

## Abbreviations

ASMR: Age-Standardized Mortality Rate
APC: Annual Percent Change
